# Metastasis-directed therapy in castration-refractory prostate cancer (MEDCARE): a non-randomized phase 2 trial

**DOI:** 10.1186/s12885-020-06853-x

**Published:** 2020-05-24

**Authors:** Charlien Berghen, Steven Joniau, Kato Rans, Gaëtan Devos, Kenneth Poels, Koen Slabbaert, Herlinde Dumez, Maarten Albersen, Karolien Goffin, Karin Haustermans, Gert De Meerleer

**Affiliations:** 1 Department of Radiation Oncology, University Hospitals Leuven, Leuven, Belgium; 2 Department of Urology, University Hospitals Leuven, Leuven, Belgium; 3 Department of Urology, RZ Tienen, Tienen, Belgium; 4 Department of Medical Oncology, University Hospitals Leuven, Leuven, Belgium; 5 Department of Nuclear Medicine and Molecular Imaging, University Hospitals Leuven, Leuven, Belgium

**Keywords:** Oligoprogression, Prostate cancer, Stereotactic body radiotherapy, Metastasectomy, Lymph node dissection, Next-line systemic treatment, Castration-refractory prostate cancer, PSMA PET-CT

## Abstract

**Background:**

Patients diagnosed with metastatic castration-refractory prostate cancer (mCRPC) rely on a limited number of therapeutic agents resulting in a median survival of 2–3 years. A subgroup of those patients with mCRPC presents with oligoprogressive disease, with a limited number of progressive lesions while other metastases are still controlled by ongoing systemic treatment.

**Methods:**

In this single arm prospective phase II trial, we aim to include 18 patients with oligoprogressive mCRPC (1–3 metastases and/or local recurrence) who will be treated with metastasis-directed therapy to all visible progressive lesions. Progression is based on conventional imaging, as the use of PSMA PET-CT is considered investigational. However all patients will undergo PSMA PET-CT and the images will be blinded until progression. Primary endpoint is the postponement of the start of next-line systemic treatment (NEST) and the additional clinical value of PSMA PET-CT. Recruitment of patients for this trial started in January 2020 and will be completed approximately by December 2020.

**Discussion:**

In this phase 2 trial on oligoprogressive mCRPC, we will investigate the benefit of progression-directed therapy while continuing ongoing systemic treatment. We hypothesize that progression-directed therapy (PDT) with surgery or stereotactic body radiation therapy for these oligoprogressive lesions will postpone the start of next-line systemic treatment and therefore serve as a new or add-on therapy in the spectrum of treatments available for mCRPC. The results of this trial will serve as guidance for a later randomized phase 3 trial. All participants are given an information sheet and are required to give written informed consent. Results will be published in a peer-reviewed journal.

**Trial registration:**

This study is registered at ClinicalTrials.gov: NCT04222634 (December 18th 2019).

## Background

In patients with metastatic prostate cancer who receive androgen deprivation therapy (ADT), the sensitivity to castration will eventually disappear due to the out-selection of castration-refractory clones. This will lead to the stage of metastatic castration-refractory prostate cancer (mCRPC) [1], which is incurable and results in a median survival of 2–3 years [2–6]. Treatment options of mCRPC include several systemic agents, such as androgen receptor targeted agents (ARTA), chemotherapy (docetaxel, cabazitaxel) and bone-targeting agents (radium-223). Clinical and iconographic progression and to a lesser extent biochemical progression, traditionally implies a switch to a **NE**xt line **S**ystemic **T**reatment (NEST). Within this group of progressive patients, there is a subgroup showing oligoprogression, typically defined as the progression of up to 3 lesions, including both metastatic and/or local relapse. Oligoprogression reflects a heterogeneous treatment response, which, in its turn, reflects the heterogeneity of the clonogenic cells that give rise to mCRPC [7]. Preliminary data, mainly from retrospective studies suggested that progression-directed therapy (PDT) to these oligoprogressive lesions allows patients to remain on their current systemic therapy, thereby delaying the need for NEST. Preliminary data support this hypothesis [8–19]. Our research group recently published promising results on the use of PDT in this setting, with a next-line systemic treatment-free survival (NEST-FS) of 21 months [20].

In order to consolidate the retrospective results of PDT in oligoprogressive mCRPC patients, we initiated a prospective phase 2 trial (MEDCARE), of which the design is presented in this manuscript.

MEDCARE (**ME**tastatis-**D**irected therapy in **CA**stration-**RE**fractory disease) is a non-randomized phase 2 trial that addresses the following research question: is PDT able to postpone the need for NEST in mCRPC patients presenting with oligometastatic and/or local progression?

In mCRPC patients, standard imaging consists of bone scan and CT-scan of thorax and abdomen [21]. Novel imaging techniques such as PSMA (prostate specific membrane antigen) PET-CT are becoming widespread available. While in patients with biochemical recurrence after primary treatment (castration sensitive patients) guidelines advise the use of PSMA PET-CT to evaluate clinical progression [21], the exact role of PSMA PET-CT in mCRPC is still unknown. Therefore, we will also address this secondary research question: what is the clinical additional value of PSMA PET-CT concerning NEST-free survival (NEST-FS) in mCRPC setting?

The results from this trial that tackle both the issue of PDT and the use of PSMA PET-CT in the treatment of oligoprogressive mCRPC, will serve as a solid guidance for a randomized phase 3 trial in the near future.

## Methods/design: the aim/design and setting of the study, the characteristics of the participants, a clear description of all processes, interventions and comparisons, type of statistical analysis

This study is approved by the Ethics committee of the Leuven University Hospitals (EC2019/S63177) and is registered on clinicaltrials.gov (NCT04222634).

MEDCARE will recruit patients presenting with oligoprogressive mCRPC. mCRPC is defined as the presence of biochemical and clinical progression of metastatic prostate cancer patients who have serum testosterone castrate levels (testosterone level < 50 ng/dl or 1.7 nmol/l). Oligoprogression is defined as the progression of at most 3 lesions, including both metastatic and/or local.

### Inclusion and exclusion criteria

Inclusion Criteria

- Histologically proven initial diagnosis of adenocarcinoma of the prostate.
- mCRPC setting, with testosterone level < 50 ng/dl or 1.7 nmol/l [22].
- Oligoprogressive disease, defined as the progression of pre-existing metastatic disease, and/or the appearance of new metastases and/or the appearance of a local relapse with a maximum of 3 lesions in total. Oligoprogression is diagnosed on conventional imaging with CT and bone scan.
- Patients currently treated with ADT, whether or not combined with another systemic treatment such as ARTA, chemotherapy or radium-223. Denosumab is allowed but not considered as second-line systemic treatment.
- Prior treatment of the primary tumor by radiotherapy or surgery. If the primary tumour is not treated, local therapy should be added to the treatment.
- WHO performance status 0–2.
- Age > = 18 years old.
- Absence of any psychological, familial, social or geographical condition potentially hampering compliance with the study protocol and/or follow-up schedule. Those conditions should be discussed with the patient before registration in the trial.
- Patient should be presented at the multidisciplinary tumour board of the local hospital in which the therapy will be given.
- Before patient registration/randomization, written informed consent must be given according to ICH/GCO and national/local regulations.

Exclusion Criteria

- Serum testosterone level > 50 ng/ml or > 1.7 nmol/l.
- Presence of polyprogression, defined as more than 3 progressive/new metastatic lesions and/or local recurrence (which counts for 1 lesion). Polyprogression is diagnosed on conventional imaging with CT and bone scan.
- Active malignancy other than prostate cancer that can potentially interfere with the interpretation of the trial.
- Previous treatments (RT, surgery) or comorbidities making new treatment with PDT impossible.
- Disorder precluding understanding of trial information or informed consent or signing informed consent.

### Interventions

All patients eligible for the trial will be discussed at the multidisciplinary urology tumour board. Inclusion in the study will be allowed only after approval of this board. The choice between SBRT/fractionated radiotherapy or surgery (see below) will also be decided on this multidisciplinary urology tumour board and will be based on the localization and size of the metastases, the nearby organ-at-risk, technical feasibility, any previous treatment as well as patient medical history and preferences.

### Surgery

In the case of pelvic lymph node metastasis, a template-based pelvic lymph node dissection (pLND) is preferred above removal of the suspicious LN only. pLND is defined as the removal of lymph nodes distal to the aortic bifurcation (obturator, internal and external iliac, common iliac, presacral and/or perirectal). In case of retroperitoneal metastasis, retroperitoneal LND is defined as the removal of lymph nodes above the aortic bifurcation, with the upper level of at least 1 cm above the most cranial lesion and preferentially till the level of the renal vasculature. In case of bone metastasis or visceral metastasis, resection will be in agreement and performed according to the expertise of the surgeon specialized for the respective location of metastasis.

### Radiotherapy

#### SBRT for oligoprogressive metastases

All patients will receive a CT simulation in supine position with 1 mm CT slice thickness through the oligoprogressive site(s) and neighbouring organs at risk. The isocentre will be placed in the centre of the lesion. In order to increase patient comfort and stability, different support devices can be used depending on the location of the oligoprogression. If 2 or 3 lesions are to be treated at the same time, there will be a separate isocentre per lesion whenever possible.

The oligoprogressive metastatic lesion(s), as visible on CT-scan, bone scan and MRI if available (preferable in case of vertebral metastases and/or local recurrence), will serve as the gross target volume (GTV). The GTV will be expanded with a 3–5 mm margin to create the Planning Target Volume (PTV). This margin accounts for organ motion and setup error.

Using intensity-modulated arc therapy (IMAT) or volumetric arc therapy (VMAT), the aim is to prescribe a biologically equivalent dose (BED) of at least 100 Gy using an alpha over beta ratio of 3 Gy. Typically a total dose of 30 Gy will be prescribed in 3 fractions of 10 Gy. Treatment will be prescribed to the periphery of the target and (80% of the dose (=30 Gy) should cover 90% of the PTV). Dose constraints for organ at risks (OAR) will be in accordance the AAPM Task Group 101 report [23]. In any case, the dose constraints for OAR have priority above the prescription dose to the PTV (see above).

Treatment will be delivered using 6 to 10-MV photons from a linear accelerator. Image-guided radiotherapy is obligatory and will be performed using daily cone-beam CT. In case of multiple isocentres, every isocentre will be verified separately. Fractions will be separated >/= 48 h and < 72 h.

#### Local recurrence

Local recurrence is diagnosed using multiparametric MRI (mpMRI) in all cases (see above).

In case the prostate and seminal vesicles are still in situ, a dose of 56 Gy in 16 fractions will be prescribed to the PTV of the prostate and seminal vesicles. The local recurrence, as visible on mpMRI, will receive a simultaneous integrated boost [24] to a dose as high as clinical achievable. Treatment will be delivered using 6 to 10-MV photons from a linear accelerator. Image-guided radiotherapy is obligatory and will be performed using daily cone-beam CT [25].

In case the local relapse is present in the post-prostatectomy situation, the PTV of the prostate bed and seminal vesicle bed will be treated to 70 Gy to be delivered in 35 fractions. The local recurrence, as visible on mpMRI, will receive a simultaneous integrated boost to a dose as high as clinical achievable. Biopsy of the recurrence is allowed if decided by the multidisciplinary board. Image-guided radiotherapy is obligatory and will be performed using daily cone-beam [25].

### Trial procedures

After being found eligible for participation to the study and prior to treatment, patients will receive a standard clinical examination and laboratory analysis including PSA, testosterone, peripheral blood count with formula, kidney and liver function with alkaline phosphatase measurement. A baseline Quality-of-life questionnaire (QLQ-30, QLQ-PR-25 and EQ-5D) [26–28] as well as baseline toxicity scoring will be performed [29, 30] after signature of the informed consent. Prior to start, conventional imaging existing of CT thorax/abdomen, bone scintigraphy (and MRI, if needed), will be completed by investigational imaging with ^18^F-PSMA PET-CT. In order not to interfere with treatment decisions, the results of the PSMA PET-CT will be blinded for the patients and investigators until progression. During fractionated radiotherapy, the physician will see patients on a weekly basis during the entire course of the treatment. In case of SBRT, patients will be seen on the last day of treatment. For all patients, at the last day of radiation, or at the day of surgery, patients will receive standard clinical examination and laboratory analysis and registration of the toxicity scoring and completion of the QOL questionnaires. After treatment, a member of the multidisciplinary urology-oncology team will see patients in follow-up visits according to a fixed schedule. There are 5 follow-up visits scheduled in year 1 (month 1 and thereafter on 3-monthly basis) and 2 visits in year 2–5 (6-monthly basis) unless clinical evolution requires more frequent follow-up visits. At each visit, a history and physical examination will be conducted with recording of the toxicity. Routine laboratory tests will be repeated. The QOL questionnaires will be concluded at each visit. Restaging with CT scan and bone scintigraphy will be performed in case of symptomatic progression and/or PSA progression, with an increase of PSA confirmed in a second blood test three weeks after the first blood test. In any case radiographic disease will be evaluated by standard imaging in month 6, month 12, month 18 and month 24. When progressive disease is confirmed as polyprogression (> 3 progressive or new lesions) or when symptomatic disease demanding new systemic therapy, a second ^18^F-PSMA PET-CT will be performed. The results of the first ^18^F-PSMA PET-CT will be unblinded at progression. A flow diagram of the trial with a summary of the main trial activities can be found in Table 1.

**Table 1:** Study visits and procedures

	Screen visit	Pre MDT (SBRT or surgery)	Last day of MDT (SBRT or surgery)	Follow-up M1, M3, M6, M9, M12 M15, M18, M21, M24				
Informed consent	X							
Clinical examination	X		X	X	X	X	X	X
Laboratory analysis	X		X	X	X	X	X	X
Registration of pre-treatment morbidities		X						
Registration of QOL using validated questionnaires		X		X	X	X	X	X
Registration of MDT induced toxicity (CTCAE for radiotherapy or Clavien-Dindo for metastasectomy)			X	X	X	X	X	X
Standard imaging (after MTB decision)	X	X (imaging at month M6, M12, M18 and M24 and at any time in case of PSA progression or symptoms)						
Investigational Imaging (^18^F PSMA-PET CT)	X (Blinded)	X (in case of PSA rise (confirmed once), radiographic progression on standard imaging or symptoms)						

X reflects the timepoint of a certain assessment

*MDT* metastasis-directed therapy, *SBRT* stereotactic body radiation therapy, *QOL* quality of life, *CTCAE* Common Toxicity Criteria of Adverse Effects, *MTB* multidisciplinary tumor board, *PSA* prostate specific antigen, *PSMA PET-CT* prostate-specific membrane antigen positron emission tomography-computed tomography

### Endpoints

#### Primary endpoint

- NEST-FS.

The aim of the study is to evaluate whether PDT is able to postpone NEST in mCRPC patients who present with oligoprogressive disease. PDT can consist of stereotactic body radiotherapy (SBRT) or metastasectomy in case of metastatic progression or fractionated radiotherapy in case of local progression. NEST-FS will be calculated from the last day of treatment in case of SBRT/fractionated radiotherapy or from the day of metastasectomy, until the first day of NEST or death. NEST will be initiated in case of polyprogression (> 3 lesions/local progression) or local progression of a treated lesion as depicted on standard imaging. The decision to initiate NEST will be taken on the multidisciplinary urological tumor board. This individual decision to continue or discontinue systemic treatment will be reported with date and specific reason. Therapy may be continued if progression occurs in ≤3 progressive lesions and a new treatment with MDT is provided.

b. Additional value of PSMA PET-CT.

All patients will be asked to undergo a ^18^F-PSMA PET-CT (using ^18^F-PSMA-1007) in addition to the conventional imaging (see above). Both patients and clinical investigators (CB, GDM, SJ) will be blinded for the results of this ^18^F-PSMA PET-CT until initiation of NEST. We want to evaluate if:

1. at time of PSA progression, new lesions will be visible only on PSMA PET-CT or both on conventional imaging and PSMA PET-CT.
2. new lesions on CT/ bone scan of radiographic progression were already visible as active lesions on the first PSMA PET-CT (and not on conventional imaging) at inclusion (as in the proportion of PSMA PET-CT positive lesions that are positive for new or progressive metastatic disease by bone scan/CT at time of progression following PDT)
3. active lesions visible at the first PSMA PET-CT at diagnosis of oligoprogression (and not on conventional imaging) might disappear as a consequence of the abscopal effect.

Practically, patients will receive a first PSMA PET-CT at the moment of diagnosis of oligoprogressive mCRPC. In order not to interfere with treatment decisions and evaluate the true value, this investigation will be blinded for both the patient and the clinicians until PSA-progression, radiographic progression on standard imaging or symptoms. At time of progression, a second PSMA PET-CT will be performed and the results of the first PSMA PET-CT will be un-blinded. All PSMA PET-CT scans will evaluated by an experienced nuclear medicine physician (KG). There is no blinding for the initial CT scan or bone scan nor the medical history, at time of evaluation of the PSMA PET-CT scan.

#### Secondary endpoints

- PSA response: a decline in PSA value ≥50% from baseline confirmed by a second value ≥4 weeks. Individual PSA change responses as a percentage change from baseline to week 12 will be plotted in a waterfall plot.
- Clinical progression-free survival (cPFS): progression is defined as the appearance of any recurrence (local, nodal or metastatic) on conventional imaging. Imaging is performed on pre-set time points (month 6, 12, 18 and 24) and in case of symptoms or PSA progression, which is defined as:

1. PSA progression after an initial decline in PSA: the time from start of therapy to first PSA increase that is ≥25% and ≥ 2 ng/ml above the nadir, and which is confirmed by a second value ≥3 weeks later [31].
2. In case of no decline from baseline: time to PSA progression with ≥25% increase and ≥ 2 ng/ml increase from baseline beyond 12 weeks. There will be a separate definition depending on the number of new and/or progressive lesions [31].

RECIST criteria v.1.1 will be used. Calculation for progression-free survival will start from the last day of SBRT / fractionated radiotherapy or from the day of metastasectomy until clinical progression or death.

c. Cancer-specific survival (CSS): cancer-specific survival (CSS) will be calculated from last day of treatment until prostate cancer death.
d. Overall survival (OS): overall survival (OS) will be calculated from last day of treatment until death from any cause.
e. Acute and late toxicity: acute and late toxicity as a result of radiotherapy will be scored using the Common Toxicity Criteria Version 4.0 [29]. Acute toxicity is defined as events occurring < 3 months following treatment. Late toxicity was specified as events occurring > 3 months following treatment or as an event lasting > 3 months after treatment. Surgical complications will be scored using Clavien-Dindo Classification [30]. Toxicity will be scored at every follow-up visit (see Table 1).

f. Quality-of-life scoring: quality of life scoring using the EORTC QLQ-30 [26] supplemented with QLQ-PR25 [27]. We will assess the quality-of-life-years with the EuroQOL classification system (EQ-5D) [28]. Assessments are planned at baseline, last day of treatment, and during every follow-up consultation.

g. Evaluation of change in patient management based on imaging results of ^18^F-PSMA PET-CT.

Systemic treatment will be continued. We aim to include 18 patients in 12 months with a minimal follow-up of 24 months. The aim is to define the time to next-line systemic treatment (NEST) in patients that are treated with progression-directed therapy for oligoprogressive mCRPC.

### Data analysis

#### Sample size calculation

Based on the number of eligible patients we expect to recruit 18 patients in one year, for which a follow-up of at least 24 months will be necessary (median NEST-FS in the PDT group was 21 months in our retrospective trial [20].

#### Statistical analysis

The primary endpoint NEST-FS will be calculated using Kaplan-Meier actuarial analyses. Survival times are defined from the last day of treatment until the start of next-line systemic treatment or last follow-up. Univariate and multivariate analysis will be performed according to the Cox-Regression method.

Overall survival, cancer-specific survival and progression-free survival will be evaluated in the same way it is the case for the primary endpoint. The incidence of acute and late toxicity will be recorded with actuarial risk estimates for developing acute and late toxicity calculated using Kaplan-Meier analysis. Statistical analysis will be performed using SPSS (v.25). We will perform a descriptive analysis of the lesion detections and potential differences of the PSMA PET-CT scans in comparison to the conventional imaging. Of particular interest is the question whether PSMA PET positive lesions, which are not detected on conventional imaging, and therefore not treated, are the drivers of progression.

## Conclusion

This protocol describes the design of a non-randomized phase II trial to evaluate the effectiveness of MDT in mCRPC presenting with oligoprogression. The results of this trial will hopefully provide a sound basis for a prospective randomized phase 3 study.

## Data Availability

The datasets generated and/or analysed during the current study are not publicly available due to national privacy policy restrictions (they contain information that could compromise research participant privacy), but are available from the corresponding author on reasonable request.

## Abbreviations

AAPM: American Association of Physicists in Medicine
ADT: Androgen deprivation therapy
ARTA: Androgen receptor targeted agents
BED: Biologically equivalent dose
cPFS: Clinical progression-free survival
CSS: Cancer-specific survival
CTCAE: Common Toxicity Criteria of Adverse Effects
GTV: Gross target volume
ICH/GCP: International Conference on Harmonisation / Good Clinical Practice
IMAT: Intensity-modulated arc therapy
mCRPC: Metastatic castration-refractory prostate cancer
MDT: Metastasis-directed therapy
MEDCARE: Metastatic-Directed therapy in CAstration-REfractory prostate cancer
mpMRI: multiparametric magnetic resonance imaging
NEST: Next-line systemic therapy
NEST-FS: Next-line systemic therapy-free survival
OAR: Organ at risk
OS: Overall survival
PDT: Progression-directed therapy
PTV: Planning target volume
QOL: Quality of life
RT: Radiotherapy
SPECT-CT: Single-photon emission computerized tomography-computed tomography
SPSS: Statistical Package for the Social Sciences
PSMA PET-CT: prostate-specific membrane antigen positron emission tomography-computed tomography
SBRT: Stereotactic body radiation therapy
VMAT: Volumetric arc therapy
WHO: World health organisation
